# Long COVID incidence in adults and children between 2020 and 2023: a real-world data study from the RECOVER Initiative

**DOI:** 10.21203/rs.3.rs-4124710/v1

**Published:** 2024-04-26

**Authors:** Hannah Mandel, Yun Yoo, Andrea Allen, Sajjad Abedian, Zoe Verzani, Elizabeth Karlson, Lawrence Kleinman, Praveen Mudumbi, Carlos Oliveira, Jennifer Muszynski, Rachel Gross, Thomas Carton, C. Kim, Emily Taylor, Heekyong Park, Jasmin Divers, J. Kelly, Jonathan Arnold, Carol Geary, Chengxi Zang, Kelan Tantisira, Kyung Rhee, Michael Koropsak, Sindhu Mohandas, Andrew Vasey, Mark Weiner, Abu Mosa, Melissa Haendel, Christopher Chute, Shawn Murphy, Lisa O’Brien, Jacqueline Szmuszkovicz, Nicholas Güthe, Jorge Santana, Aliva De, Amanda Bogie, Katia Halabi, Lathika Mohanraj, Patricia Kinser, Samuel Packard, Katherine Tuttle, Lorna Thorpe, Richard Moffitt

**Affiliations:** New York University Grossman School of Medicine; Emory University; Children’s Hospital of Philadelphia; Weill Cornell Medicine; Weill Cornell Medical College; Brigham and Women’s Hospital; Rutgers Robert Wood Johnson Medical School; New York University Grossman School of Medicine; Yale University; Nationwide Children’s Hospital; New York University Grossman School of Medicine; Louisiana Public Health Institute; RECOVER Patient, Caregiver, or Community Advocate Representative; RECOVER Patient, Caregiver, or Community Advocate Representative; Mass General Brigham; New York University, USA; University of California, San Francisco; University of Pittsburgh School of Medicine; University of Nebraska Medical Center; Weill Cornell Medicine; Division of Respiratory Medicine, Department of Pediatrics, University of California San Diego, San Diego; University of California, San Diego; Weill Cornell Medical College; Children’s Hospital Los Angeles/University of Southern California; University of Nebraska Medical Center; Weill Cornell Medicine; University of Missouri School of Medicine; University of Colorado Anschutz Medical Campus; Johns Hopkins University; Massachusetts General Hospital; RECOVER Patient, Caregiver, or Community Advocate Representative; Childrens Hospital Los Angeles; RECOVER Patient, Caregiver, or Community Advocate Representative; University of Puerto Rico; Columbia University Irving Medical Center; University of Oklahoma Health Sciences Center; Nationwide Children’s Hospital; Virginia Commonwealth University School of Nursing; Virginia Commonwealth University School of Nursing; Columbia University Mailman School of Public Health; University of Washington; New York University Grossman School of Medicine; Emory University

## Abstract

Estimates of post-acute sequelae of SARS-CoV-2 infection (PASC) incidence, also known as Long COVID, have varied across studies and changed over time. We estimated PASC incidence among adult and pediatric populations in three nationwide research networks of electronic health records (EHR) participating in the RECOVER Initiative using different classification algorithms (computable phenotypes). Overall, 7% of children and 8.5%–26.4% of adults developed PASC, depending on computable phenotype used. Excess incidence among SARS-CoV-2 patients was 4% in children and ranged from 4–7% among adults, representing a lower-bound incidence estimation based on two control groups - contemporary COVID-19 negative and historical patients (2019). Temporal patterns were consistent across networks, with peaks associated with introduction of new viral variants. Our findings indicate that preventing and mitigating Long COVID remains a public health priority. Examining temporal patterns and risk factors of PASC incidence informs our understanding of etiology and can improve prevention and management.

## Introduction

Post-acute sequelae of SARS-CoV-2 infection (PASC), commonly referred to as Long COVID, has emerged as a significant clinical and public health concern following the global COVID-19 pandemic.^1^ PASC refers to ongoing, intermittent, or new symptoms or conditions resulting from acute SARS-CoV-2 infection, beginning 1–3 months after onset of mild or severe COVID-19 illness.^2–4^ These prolonged effects can profoundly impact quality of life and lead to functional limitations, psychological distress, and higher numbers of healthcare visits.^5^

Characterization of the epidemiology of PASC to guide the larger public health response has remained challenging due to the wide range of symptoms. Various studies have quantified how many patients with SARS-CoV-2 infections may subsequently develop PASC, resulting in a wide range of estimates. A recent systematic review found that 45% of patients with COVID-19 developed at least one unresolved symptom, with higher prevalence among hospitalized patients; however, estimates ranged from 2–99.9% across studies.^6^ Differences in PASC definitions, data sources, study populations, and methodology have contributed to inconsistent results.^7,8^ In the absence of a gold standard, multiple case definitions for PASC have been operationalized by researchers.^3,4^

Another potential source of variation is the study period. Some studies have centered on a brief period, focusing on symptom prevalence over a specified time after acute infection.^9–12^ Fewer studies have explored fluctuations in PASC frequency over variant waves. Some of these suggest that risk of PASC has declined with recent COVID-19 variants,^13–19^ while others have found similar or higher levels of risk with Omicron.^20,21^ Larger studies are needed to systematically elucidate whether variations in PASC incidence are associated with variant differences, and whether incidence has declined after introduction of acute COVID-19 treatments, vaccination efforts, and immunity supplied by prior infection.

As part of the National Institutes of Health (NIH)-funded REsearching COVID to Enhance Recovery (RECOVER) Initiative, we leveraged electronic health record (EHR) data from three large EHR-based research networks to estimate PASC incidence. Our analysis considered demographic factors, including age, sex, race, ethnicity, and residential setting. We also examined how temporal trends varied across viral variant waves. Given the lack of a unified definition of PASC, our analyses leveraged three definitions developed by each network. By exploring patterns of PASC across networks over time using a range of definitions, our study contributes to the growing body of knowledge on sequelae of SARS-CoV-2 infection to inform ongoing healthcare planning, intervention strategies, and patient care.

## Methods

### Participating Institutions

RECOVER is a large NIH-funded initiative designed to investigate the long-term effects of COVID-19, including EHR-based research that comprises three participating research networks: the National COVID Cohort Collaborative (N3C), a centralized repository of COVID-19 positive patients matched to non-infected controls;^22^ the National Patient-Centered Clinical Research Network (PCORnet); and PEDSnet, a pediatric learning health system within PCORnet. Cross-network studies are coordinated by a Clinical Science Core at NYU Langone Health. For these analyses, N3C selected 18,406,079 unique patient records across 68 sites, PCORnet selected 18,893,900 records across 27 sites, and PEDSnet selected 8,200,977 records across 38 sites. See more information about data sources in Supplement eMethods.

### Study Population

We identified adult and pediatric patients with evidence of SARS-CoV-2, including a: (1) positive polymerase chain reaction (PCR) or antigen test; (2) International Classification of Diseases, Tenth Revision (ICD-10) diagnosis code representing COVID-19 (U07.1); (3) prescription for nirmatrelvir/ritonavir (Paxlovid) or Remdesivir; (4) post-acute evidence of SARS-CoV-2 infection, including ICD-10 codes M35.81 (Multisystem Inflammatory Syndrome in Children, or MIS-C), U09.9 (PASC), or B94.8 (Sequelae of other specified infectious and parasitic diseases),^23^ and pediatric positive nucleocapsid IgG test results. We defined the index event as the first documented evidence, and for patients with post-acute evidence only we imputed index events as 59 days prior to the earliest, placing the window for PASC onset after the acute infection period but minimizing possibility of the window being associated with reinfection.^24^

Pediatric patients were included if ≤21 years old at index event (PEDSnet), and adult patients were included if ≥22 (N3C, PCORnet). Patients with an index event between March 2020 and February 2023 were included, leaving at least six months before the end of the study period (September 2023) to allow time for PASC to be documented and mitigate lags in data reporting. We also required patients to have at least one visit within the health system prior to index event, and at least one follow-up visit occurring during the study period and 90 or more days after the index event. See patient attrition details in Supplement eFigure 2.

### Outcomes

EHR networks have iteratively developed separate PASC definitions using different populations and technical infrastructures. Given the lack of a gold standard, we saw this as an opportunity to look across definitions. Each network applied its own computable phenotype for probable PASC (herein ‘PASC’). N3C’s definition identified a) earliest documentation of a U09.9 / B94.8 diagnosis code or b) patients with predicted PASC by a machine-learning based algorithm trained on patients with a U09.9 diagnosis.^25^ PCORnet and PEDSnet applied rules-based definitions based on a combination of clinical input and data-driven analysis of new-onset diagnoses more common in patients with COVID-19 than without.^26–28^ Generally, PCORnet classified patients based on the presence of a U09.9 or B94.8 code, or at least one incident PASC diagnosis.^26,29^ PEDSnet classified patients based on diagnoses (one U09.9, B94.8, M35.81 code, or two or more PASC-associated features at least 28 days apart). See Supplement eMethods for network-specific descriptions.

All networks examined the proportion of COVID-19 patients developing PASC within 30–180 days after the index event (incidence proportion) and defined PASC onset as the earliest date that the computable phenotype identified PASC. Rates were presented as percentages (per 100 COVID-19 positive patients) over the entire study timeframe and by month or variant wave.

### Timeframes and covariates

We defined the following time periods as distinct waves for COVID-19 variants: Ancestral (March-December 2020), Alpha (January-May 2021), and Delta (June-November 2021). We identified three separate Omicron waves: BA.1.1 - BA.2 (December 2021-April 2022), BA.2.12.1 - BA.5 (May-November 2022), and BQ.1.1 - XBB.1.5 (December 2022-August 2023).^30^

We also explored incidence by patient age, sex, race/ethnicity (categorized as either Hispanic; non-Hispanic Asian, Black, other, or white; or missing/unknown), and rurality. Rurality was assessed by linking patient 5-digit ZIP codes to an urban commuting area (RUCA) classification (metropolitan, micropolitan, small town, or rural).^31^ Finally, we examined incidence by 1) severity of infection, 2) documentation of vaccination or treatment for COVID-19, and 3) pre-existing conditions prior to index event (see Supplement eMethods).

### Statistical analysis

We summarized categorical data as counts and percentages and described continuous data as mean values with standard deviations (SDs) or median values with interquartile ranges (IQRs). We analyzed 180-day incidence proportions using the beta distribution to calculate 95% confidence intervals (CIs) and represented findings visually as time-series plots and heatmaps, including summing patients with PASC by month of PASC onset. Unadjusted and adjusted hazard ratios with 95% CIs were generated using multivariable Cox Proportional Hazards regression models with PASC onset as an endpoint and presented by age group, sex, race/ethnicity, pre-existing conditions, rurality, COVID severity, vaccination status, and index month. P-values <0.05 were considered statistically significant. All analysis and visualizations were created using R, including ggplot2, survival, and survminer packages.

### Control group analysis

Because many PASC symptoms are non-specific, we compared PASC-positivity within a COVID test-negative control group against COVID-19 positive patients within the same period. “Negative” controls were required to have a negative PCR or antigen test within the first five months of 2021 (when testing was available but before widespread home testing), and no evidence of COVID-19 prior to or within 180 days afterwards. We included patients if their first documented negative test fell within the period and considered this the index event. We repeated this approach using a “historical” control group selected from the first five months of 2019 to estimate how many patients would be identified as PASC-positive using our algorithms during that period, selecting the first visit within that timeframe as the index event. To calculate ‘excess incidence’ for the SARS-CoV-2 study population, we considered the incidence among COVID-19 negative outpatients to approximate a baseline (“COVID-free”) burden of PASC-like sequelae and subtracted this from the overall incidence among COVID-19 positive patients.

## Results

In total, 5,445,776 COVID-19 positive patients were identified: 3,736,427 adults within N3C (mean age 51 years, SD 17; 61% female), 1,126,467 adults within PCORnet (mean age 53 years, SD 17; 62% female), and 582,882 pediatric patients within PEDSnet (mean age 9 years, SD 7; 50% female) (Supplement **eTable 1**). Most adults (58–67%) and 42% of pediatric patients were non-Hispanic white. The majority resided within urban settings (N3C: 71%, PCORnet: 72%, PEDSnet: 60%). Severity of COVID-19 index infections was similar across networks. Few patients were hospitalized for their index events (with or without ICU-level care: N3C: 6%, PCORnet: 8%, PEDSnet: 2%). Few (0.4%) children had evidence of MIS-C (data not shown).

Between 7% and 26% of patients were identified as having PASC, with the PCORnet estimation being the highest (PCORnet: 26.4%, N3C: 8.5%, PEDSnet: 7.0%) (Table 1). Of these, less than 10% lacked data on acute infection timing and had index dates imputed (PEDSnet: 9.6%, N3C: 8.9%, PCORnet: 6.1%). Patients with PASC were more likely to have been hospitalized for COVID-19 than those without, and to have a higher burden of pre-existing conditions, particularly in N3C. Nearly all children with MIS-C (95.5%) were categorized as having PASC (data not shown).

**Figure 1:**
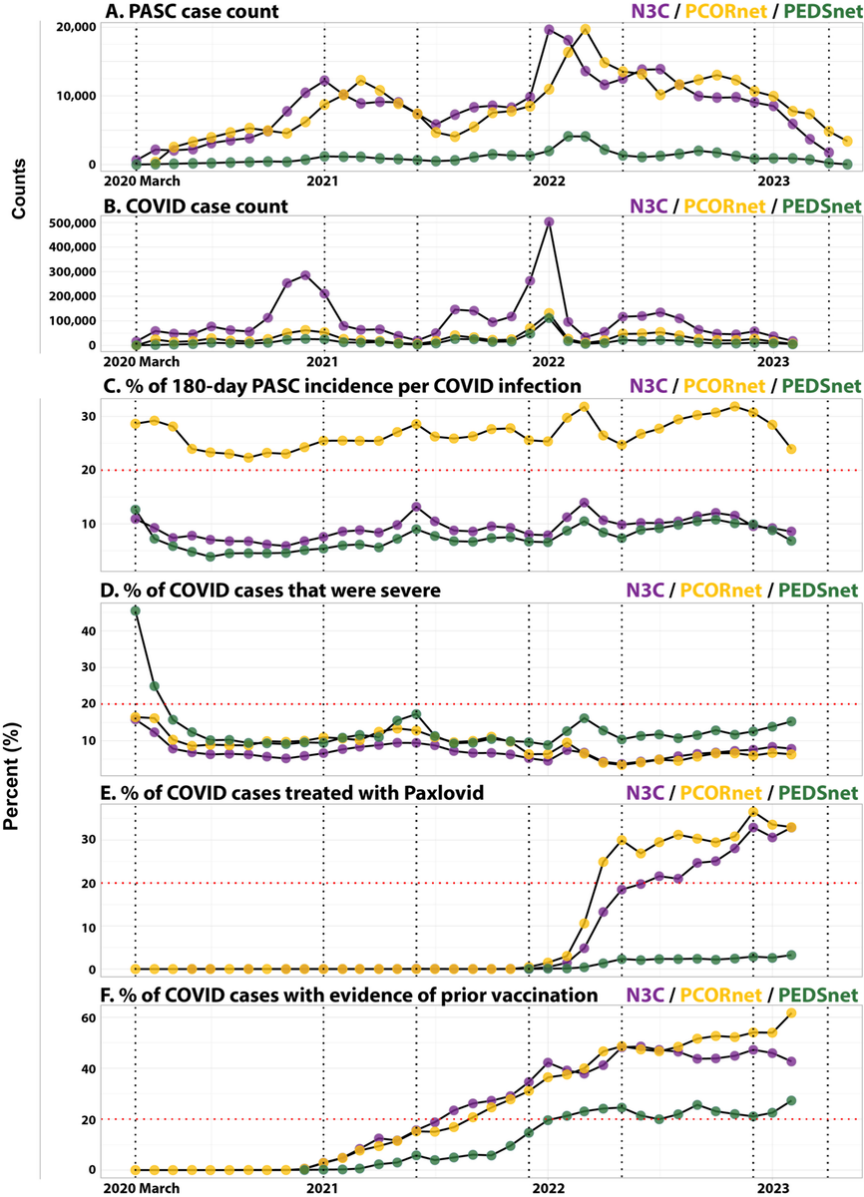
Proportion of patients with PASC and associated variables over time PASC incidence and associated variables by month. Each patient was counted as having COVID-19 once, and COVID-19 index dates were graphed on the x-axis for panels b-f. Dotted red line provides a 20% benchmark across differently sized axes. A**, Count of PASC cases**, with the x-axis representing the date of PASC onset. **B, Count of COVID-19 index cases** each month. **C, Percentage of patients with PASC**. Percentage was calculated using the monthly COVID-19 case count as the denominator, and number of patients with PASC within 180 days of the COVID-19 index date as the numerator. **D, Percentage of COVID-19 cases** hospitalized for index infection. Percentage was calculated using the monthly COVID-19 case count as the denominator, and the number of hospitalized cases with or without ICU-level care as the numerator. **E, Percentage of COVID-19 cases treated with Paxlovid**. Percentage was calculated using the monthly COVID-19 case count as the denominator, and the number of hospitalized cases with or without ICU-level care as the numerator. This excludes 19 N3C sites not providing any data around Paxlovid orders. **F, Percentage of COVID-19 cases with prior vaccination**. Percentage was calculated using the monthly COVID-19 case count as the denominator, and the number of cases with evidence of vaccination prior to the index event as the numerator.

Because most patients had COVID-19 prior to widespread use of Paxlovid around Spring 2022,^32^ few had Paxlovid prescribed for their index infection (N3C: 4%, PCORnet: 9%, PEDSnet: 1%) (**eTable 1**). In a sub-analysis over June 2022-September 2023, excluding 19 sites not reporting Paxlovid data to N3C, monthly percentages of adults treated with Paxlovid surpassed 20% (Figure 1E). The proportion of patients developing PASC was similar for treated and untreated patients (Table 1).

Documented vaccinations were consistently low across adult EHR networks, with 24% of N3C patients and 26% of PCORnet patients having at least one COVID-19 vaccination prior to index event (Figure 1F, **eTable 1)**. This number was lower (12%) for PEDSnet patients. In unadjusted analyses, proportions of patients developing PASC were similar by vaccination status (Table 1).

### Monthly trends for COVID-19 and PASC cases

Figure 1 shows the occurrence of COVID-19 and PASC from March 2020 to February 2023, together with changes in related covariates. Monthly count patterns were concordant across PCORnet and N3C, including spikes in cases around the beginning of 2022 (Figure 1A), while PASC case counts among children were lower. COVID-19 case counts were also closely aligned across networks (Figure 1B), including surges in cases following the onset of new variant waves. While absolute PASC incidence proportions differed, temporal patterns were strikingly similar (Figure 1C). A decline in incidence towards the end of the study period was noted across networks. However, analysis of source data newer than those used for this manuscript confirms that this apparent decline is unreliable (data not shown), and likely an artifact of incomplete follow-up times for some of our population due to heterogeneous follow-up and reporting schedules across sites. Monthly percentages of severe COVID-19 cases were concordant (Figure 1D).

**Figure 2:**
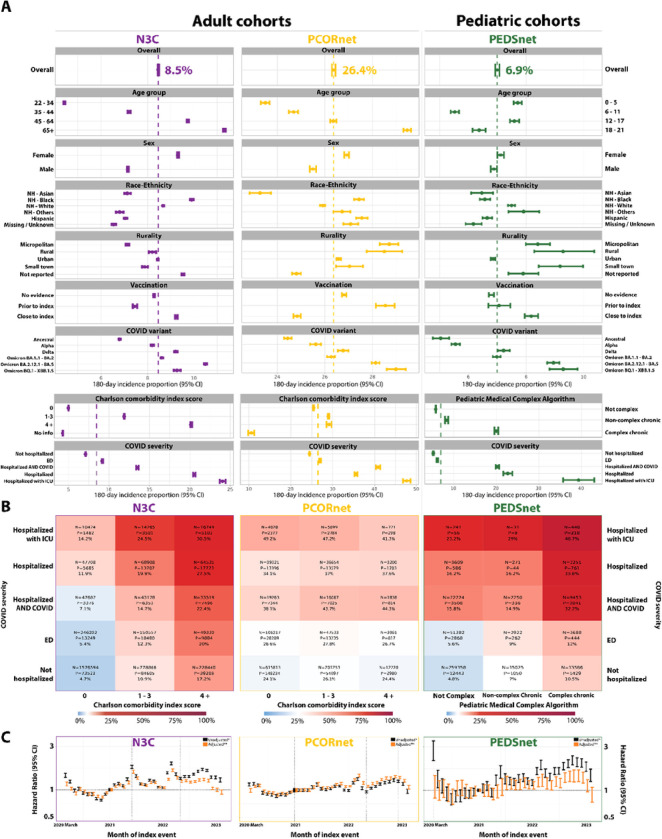
PASC incidence proportion by patient subpopulation and adjusted monthly relative hazards. **A, Univariate analysis.** Incidence of PASC, calculated as the proportion of COVID-19 cases who developed PASC within 180 days of index infection. 95% Confidence Intervals are provided. Patient characteristics align with categories described in Table 1. Vertical dotted lines represent overall PASC incidence proportion for each network. **B, Two-Dimensional Heatmap.** Heatmaps represent the proportion of COVID-19 positive patients who developed PASC. Percentages are stratified by COVID-19 severity and patient pre-existing conditions. N represents the number of patients within the group. P represents the number of PASC patients within the group. Heatmap scales are based on the percentage of PASC patients from each network, from 0% (blue) through 100% (red). The midpoint (white) of the scale represents the overall PASC rate from each network. Values three or more times greater than the overall PASC rate were colored red. **C, Risk of PASC over time compared to January 2021**. Multivariable hazard ratios for incident PASC per month. Hazard ratios were generated by a multivariable Cox Proportional Hazards regression model, and are presented unadjusted (black) and adjusted (orange) for age group, sex, race/ethnicity, pre-existing conditions, rurality, COVID-19 severity, vaccination status, and month of the index event. 95% Confidence Intervals are provided.

Across networks, PASC incidence was higher for female patients (Figure 2A) and, among adults, for older age groups and Black patients. Notably, incidence increased across variant waves: it was lowest during the Ancestral and Alpha waves, followed by Delta and early Omicron, and was highest for recent Omicron variants. Incidence was frequently greater with higher Charlson Comorbidity Index (CCI) scores, Pediatric Medical Complexity Algorithm (PMCA) category, and COVID-19 severity (Figure 2B).

### Multivariable analysis

We constructed time-to-PASC multivariable Cox regression models (Figure 2C). Unadjusted and adjusted hazard ratios (HR and aHRs; 95% CI) for PASC incidence within 180 days were summarized over the study period (Table 2). Among adults, older age groups had a significantly higher PASC risk, with highest risk among patients 65 and older (N3C HR: 2.66 (2.63–2.69), PCORnet HR: 1.31 (1.30–1.33)). This attenuated but remained significant after adjustment (N3C aHR: 1.45 (1.43–1.47), PCORnet aHR: 1.21 (1.20–1.22)). In children, children aged 6–11 had significantly lower PASC risk than other age groups, including after adjustment. Overall, male patients had significantly lower risk of PASC than female patients (N3C HR: 0.76 (0.76–0.77), PCORnet HR: 0.94 (0.93–0.95), PEDSnet HR: 0.96 (0.93–0.98)), an association that was strengthened after adjusting for covariates.

Risk of PASC varied by race and ethnicity, with Asian patients having a slightly lower PASC risk compared to white patients (Table 2). Results were otherwise inconsistent across networks. After adjustment, adult patients with documented prior vaccination had a significantly lower risk of PASC compared to those with no evidence, with a stronger observed effect among PCORnet patients (aHR: 0.84, p<0.001) than N3C (aHR: 0.98, p<0.001). In N3C, patients vaccinated within 90 days post-index event also had slightly lower risk of PASC (aHR: 0.97, p<0.001).Prior vaccination was associated with a slightly higher risk of PASC among pediatric patients (aHR: 1.1, p<0.001).

Acute COVID-19 illness severity was the strongest predictor of PASC risk (Table 2) Patients hospitalized with ICU-level care had the highest risk, even after adjustment (N3C aHR: 2.53, p<0.001; PCORnet aHR: 2.31, p<0.001; PEDSnet aHR: 2.29, p<0.001). Burden of pre-existing conditions was also an important risk factor, especially within N3C, where patients with a CCI score of 0 had lower PASC risk than patients scoring 1–3 (aHR: 2.15, p<0.001) or 4+ (aHR: 3.38, p<0.001).

### Control group analysis

**Figure 3:**
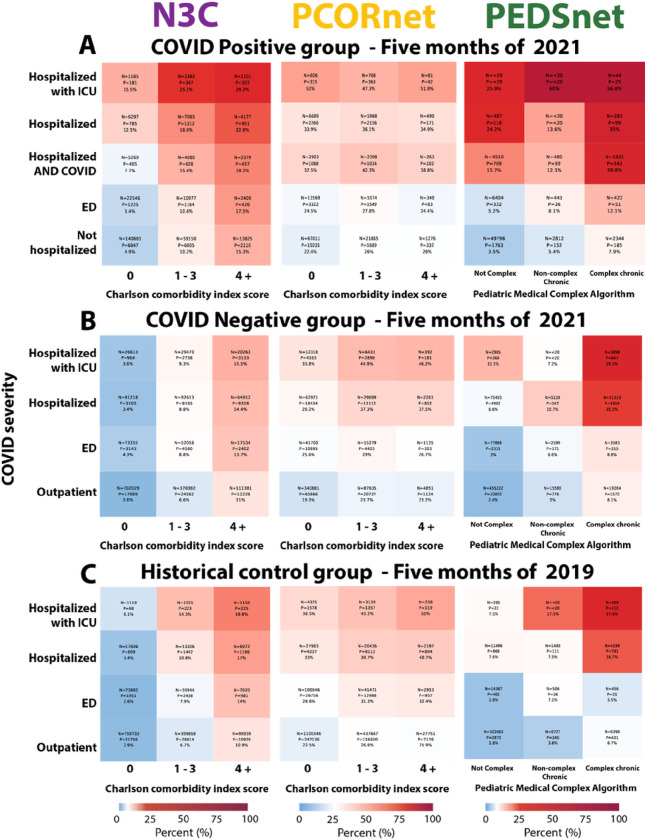
Control group analysis PASC incidence proportion, by COVID-19 severity and pre-existing condition burden, among **A, COVID-19 positive patients** identified between 1/1/2021 – 5/30/2021; **B, contemporary COVID-19 negative patients**identified between 1/1/2021 – 5/30/2021; and **C, historical control patients**identified between 1/1/2019 – 5/30/2019. Heatmaps for each group are centered (white) on the global PASC incidence estimated in that group. Lower values are shown in shades of blue, and values up to 2.5x above the global incidence are shown in shades of red. N represents the number of patients within the group. P represents the number of PASC patients within the group.

PASC-like sequelae were found in both 2019 (“historical”) and 2021 (“contemporary” COVID-19 test negative) control groups (Figure 3), but at lower levels than among COVID-19 positive patients, demonstrating the non-specific nature of symptoms associated with PASC. Rates among historical controls were higher than contemporary controls, potentially reflecting higher levels of interaction with the healthcare system in 2019 compared to 2021. Heatmaps for each control group showed the increased propensity for PASC with greater burden of pre-existing conditions.

Resulting excess PASC incidence estimates (N3C: 4.2%, PCORnet: 6.7%, PEDSnet: 4.2%) may represent a lower bound on the excess burden posed by COVID-19.

## Discussion

Our study explored crude and excess incidence of PASC among a large cohort of COVID-19-infected adults and children using nationwide EHR network data. Crude incidence estimates of PASC ranged from a low of 8.5% to a high of 26.4% in adults and was 7.0% in children. After accounting for potential background levels of PASC-like symptoms, excess incidence was estimated to be 4% in children and between 4–7% among adults. Incidence was highest in older adults, women, and patients with pre-existing comorbidities or more severe acute COVID-19 illness. While magnitude of PASC incidence varied depending on definition used, temporal patterns and risk factors were largely consistent across the EHR networks. Temporal peaks in PASC cases aligned with emergence of new viral variants, suggesting a potential association between viral dynamics and the development of PASC. Peaks aside, we did not see sizable secular decreases in PASC risk over time, suggesting that PASC remains a public health priority. However, other factors such as increasing recognition of PASC clinical features and use of U09.9 codes over time PASC over time may be influencing detection of PASC diagnoses in EHRs. Higher prevalence of PASC among older adults and individuals of certain racial or ethnic backgrounds underscores the importance of considering these factors in PASC risk stratification.

Our study relied on three distinct definitions of PASC, including a broader definition from PCORnet and a more restrictive definition from N3C. Our pediatric definition was also more restrictive (requiring two or more diagnoses indicative of PASC). Thus, we present findings as a range of potential PASC incidence estimates. Our analyses further contextualize these estimates by applying PASC definitions to two “control” groups. As expected, PASC-like sequelae were highest among COVID-19 patients, especially when considering pre-event health of patients and severity of the index encounter. We note that PASC-like symptoms may be over-represented in both control groups, which could underestimate excess incidence; contemporary controls may include patients with false-negative tests or undocumented infections, and patients had more frequent interactions with the healthcare system in 2019 compared to 2021.^33^

Consistent with other studies, our findings highlight severity of initial COVID-19 infection as a risk factor for PASC, with elevated rates of PASC among patients hospitalized during their initial COVID-19 infection,^6,34,35^ though we recognize that patients with inpatient care may receive closer follow-up, leading to more PASC diagnosis opportunity.

Consistent with a prior N3C publication,^36^ our adjusted analyses found that previously vaccinated adults had a lower risk of PASC compared to unvaccinated patients. This finding was not replicated within the pediatric cohort, perhaps because higher-risk pediatric patients are more likely to seek out vaccination. However, the sample of pediatric patients with known vaccination was also small and we were not able to differentiate unvaccinated patients from vaccinated patients lacking documentation. The impact of vaccination on PASC incidence remains an important area for investigation.

In this study, we applied a consistent analytic approach across three research networks using different PASC definitions. Despite concordance of most findings, this study had several limitations. First, diagnoses of COVID-19 and PASC may be missing in EHR data. Indeed, we suspect the high levels of PASC observed at the beginning of the pandemic reflect underdetection of mild COVID-19 while testing was rationed. Similarly, higher PASC incidence during recent variant waves could reflect growing recognition and diagnosis of PASC.^21^ Findings may also be influenced by restriction to patients with multiple healthcare encounters, underrepresentation of data from non-academic medical centers, and variation in healthcare access by gender, race/ethnicity, rurality, and other structural or social determinants of health. Our study also lacked complete data on vaccination status.

Nonetheless, our study contributes valuable insights into PASC incidence in a large cohort of COVID-19 patients. Our cross-network approach leveraged differing definitions delivered a plausible range of estimates and demonstrated remarkably similar profiles and temporal patterns of PASC incidence. Future prospective studies incorporating comprehensive assessment protocols, diverse healthcare settings, and detailed variant and vaccination data are warranted to further elucidate the complex nature of PASC and inform strategies for its prevention, management, and long-term care.

## Figures and Tables

**Table 1.** Descriptive characteristics of COVID-19-positive patients with and without PASC across three electronic health record research networks

Characteristic	N3C (N=3,736,427)		PCORnet (N= 1,126,467)		PEDSnet (N= 582,882)	
	COVID-19+ without PASC No. (%)	COVID-19+ with PASC No. (%)	COVID-19+ without PASC No. (%)	COVID-19+ with PASC No. (%)	COVID-19+ without PASC No. (%)	COVID-19+ with PASC No. (%)
**Total N**	3,424,079	316,268	829,419	297,048	542,185	40,697
**Age, mean [SD], years**	50.18 (17)	55.59 (16)	52 (17)	54 (18)	8.94 (6)	8.42 (7)
**Age groups**						
0–5	NA				201,993 (37)	17,671 (43)
6–11					132,008 (24)	7,438 (18)
12–17					141,252 (26)	10,993 (27)
18–21					66,932 (12)	4,595 (11)
22–34	795,624 (23)	36,706 (12)	167,198 (20)	51,323 (17)	NA	
35–44	1,221,080 (36)	131,495 (42)	144,817 (17)	47,446 (16)		
45–64	622,892 (18)	48,303 (15)	296,980 (36)	106,229 (36)		
65+	784,483 (23)	99,826 (32)	220,424 (27)	92,050 (31)		
**Sex**						
Female	2,062,017 (60)	211,394 (67)	508,194 (61)	187,162 (63)	272,206 (50)	20,605 (51)
Male	1,362,062 (40)	104,874 (33)	321,225 (39)	109,886 (37)	269,979 (50)	20,092 (49)
**Race/ethnicity**						
Hispanic	369,126 (11)	27,981 (9)	89,224 (11)	33,936 (11)	132,276 (24)	9,964 (25)
Non-Hispanic Asian	93,602 (3)	7,186 (2)	26,706 (3)	8,100 (3)	23,288 (4)	1,577 (4)
Non-Hispanic Black	426,623 (12)	46,859 (15)	129,257 (16)	48,860 (16)	99,326 (18)	6,973 (17)
Non-Hispanic Other	55,809 (2)	4,069 (1)	43,780 (5)	15,960 (5.4)	10,181 (2)	919 (2)
Non-Hispanic White	2,280,984 (67)	216,331 (68)	484,757 (58)	169,506 (57)	224,280 (41)	17,903 (44)
Missing / Unknown	197,935 (6)	13,842 (4)	55,695 (7)	20,686 (7)	52,834 (10)	3,361 (8)
**Rurality**						
Micropolitan	304,507 (9)	23,385 (7)	37,616 (5)	15,141 (5)	15,301 (3)	1,403 (3)
Rural	71,445 (2)	6,383 (2)	10,154 (1)	4,047 (1)	2,805 (0.5)	287 (0.7)
Small town	127,772 (4)	10,933 (3)	17,118 (2)	6,343 (2)	5,034 (0.9)	509 (1)
Urban	2,425,780 (71)	223,567 (71)	599,529 (72)	217,090 (73)	326,845 (60)	24,027 (59)
Missing	494,575 (14)	52,000 (16)	165,002 (20)	54,427 (18)	192,200 (35)	14,471 (36)
**Charlson Comorbidity Index Score**						
0	1,831,295 (53)	96,376 (30)	585,722 (71)	199,612 (67)	NA	
1–3	929,078 (27)	125376 (40)	222,672 (27)	90,716 (31)		
4+	313,154 (9)	78,830 (25)	14,487 (1.7)	5,950 (2)		
Unknown	350,552 (10)	15,686 (5)	6,538 (0.8)	770 (0.3)		
**Pediatric Medical Complexity Algorithm Category**						
None	NA				479,533 (88)	29,633 (73)
Non-complex Chronic					27,562 (5)	2,450 (6)
Complex Chronic					35,090 (7)	8,614 (21)
**COVID-19 severity**						
Not hospitalized	2,704,944 (79)	207,118 (65)	635,955 (77)	205,931 (69)	411,314 (76)	22,688 (56)
Emergency Department	425,038 (12)	42,923 (14)	114,524 (14)	42,351 (14)	82,086 (15)	5,473 (13)
Hospitalized	149,055 (4)	38,426 (12)	51,044 (6)	28,190 (10)	6,660 (1)	1,962 (5)
Hospitalized and COVID-19 positive	112,268 (3)	17,479 (6)	21,901 (3)	15,114 (5)	41,648 (8)	10,261 (25)
Hospitalized with Intensive Care Unit-level care	32,774 (0.96)	10,322 (3)	5,995 (0.7)	5,462 (2)	477 (0.1)	313 (0.8)
**Predominant variant at index event**						
Ancestral	946,001 (28)	68,886 (22)	205,107 (25)	66,317 (22)	37,114 (7)	1,858 (5)
Alpha	420,400 (12)	37,570 (12)	97,613 (12)	33,598 (11)	123,496 (23)	6,996 (17)
Delta	517,090 (15)	52,452 (17)	108,148 (13)	39,521 (13)	85,533 (16)	6,565 (16)
Omicron BA.1.1 - BA.2	868,111 (25)	81,787 (26)	193,210 (23)	68,813 (23)	178,851 (33)	13,505 (33)
Omicron BA.2.12.1 - BA.5	569,702 (17)	66,740 (21)	189,174 (23)	74,031 (25)	96,350 (18)	9,734 (24)
Omicron BQ.1.1 - XBB.1.5	102,775 (3)	10,505 (3)	36,167 (4)	14,768 (5)	20,841 (4)	2,039 (5)
**Paxlovid ordered at index event**	134,170 (4)	13,944 (4)	80,105 (10)	25,012 (8)	3,127 (0.6)	169 (0.4)
**Evidence of vaccination**						
Prior to index event	815,242 (24)	82,811 (26)	217,403 (26)	71,799 (24)	65,613 (12)	5,795 (14)
Close to index event	176,763 (5)	14,235 (5)	34,491 (4)	13,774 (5)	21,596 (4)	1,672 (4)
None documented	2,432,074 (71)	219,438 (69)	577,525 (70)	211,475 (71)	454,976 (84)	33,230 (82)

**Table 2.** Multivariable hazard ratios for development of PASC

N3C			PCORnet		PEDSnet	
	Unadjusted^a^	Adjusted^b^	Unadjusted^a^	Adjusted^b^	Unadjusted^a^	Adjusted^b^
**Age Group**						
0–5	NA				1.45^c^ (1.4 – 1.5)	1.38^c^ (1.31 – 1.44)
6–11					Ref	Ref
12–17					1.39^c^ (1.34 – 1.44)	1.24^c^ (1.18 – 1.29)
18–21					1.17^c^ (1.12 – 1.23)	1.16^c^ (1.09 – 1.23)
22–34	Ref	Ref	Ref	Ref	NA	
35–44	1.65^c^ (1.63 – 1.67)	1.51^c^ (1.49 – 1.53)	1.06^c^ (1.05 – 1.08)	1.06^c^ (1.05 – 1.07)		
45–64	2.26^c^ (2.23 – 2.28)	1.7^c^ (1.68 – 1.72)	1.15^c^ (1.14 – 1.16)	1.12^c^ (1.11 – 1.14)		
65+	2.66^c^ (2.63 – 2.69)	1.45^c^ (1.43 – 1.47)	1.31^c^ (1.30 – 1.33)	1.21^c^ (1.20 – 1.23)		
**Sex**						
Female	Ref	Ref	Ref	Ref	Ref	Ref
Male	0.76^c^ (0.76 – 0.77)	0.68^c^ (0.68 – 0.69)	0.94^c^ (0.93 – 0.95)	0.92^c^ (0.91 – 0.92)	0.96^c^ (0.93 – 0.98)	0.87^c^ (0.84 – 0.9)
**Race/Ethnicity**						
Hispanic	0.81^c^ (0.8 – 0.82)	0.88^c^ (0.86 – 0.89)	1.08^c^ (1.06 – 1.09)	1.05^c^ (1.04 – 1.07)	0.88^c^ (0.85 – 0.91)	1 (0.96 – 1.04)
Non-Hispanic Asian	0.82^c^ (0.8 – 0.84)	0.82^c^ (0.8 – 0.84)	0.88^c^ (0.86 – 0.90)	0.92^c^ (0.90 – 0.94)	0.85^c^ (0.8 – 0.91)	0.9^d^ (0.82 – 0.98)
Non-Hispanic Black	1.15^c^ (1.14 – 1.16)	0.95^c^ (0.94 – 0.96)	1.07^c^ (1.06 – 1.08)	1.03^c^ (1.02 – 1.04)	0.86^c^ (0.83 – 0.89)	0.81^c^ (0.77 – 0.84)
Non-Hispanic Other	0.78^c^ (0.76 – 0.8)	0.87^c^ (0.84 – 0.89)	1.04^c^ (1.02 – 1.06)	1.05^c^ (1.03 – 1.06)	1.05 (0.98 – 1.13)	0.95 (0.86 – 1.05)
Non-Hispanic White	Ref	Ref	Ref	Ref	Ref	Ref
Missing/Unknown	0.75^c^ (0.74 – 0.76)	0.84^c^ (0.83 – 0.86)	1.05^c^ (1.04 – 1.07)	1.05^c^ (1.03 – 1.06)	0.82^c^ (0.78 – 0.87)	0.94 (0.87 – 1.01)
**Rurality**						
Micropolitan	0.84^c^ (0.83 – 0.85)	0.82^c^ (0.81 – 0.83)	1.1^c^ (1.08 – 1.11)	1.08^c^ (1.06 – 1.10)	1.2^c^ (1.14 – 1.27)	0.99 (0.92 – 1.07)
Rural	0.97^e^ (0.94 – 0.99)	0.88^c^ (0.86 – 0.9)	1.09^c^ (1.06 – 1.12)	1.06^c^ (1.03 – 1.09)	1.33^c^ (1.18 – 1.51)	1.08 (0.93 – 1.26)
Small town	0.93^c^ (0.91 – 0.95)	0.87^c^ (0.85 – 0.89)	1.02 (1.00– 1.05)	1.01 (0.98 – 1.03)	1.38^c^ (1.26 – 1.5)	1.17^e^ (1.04 – 1.31)
Urban	Ref	Ref	Ref	Ref	Ref	Ref
Missing	1.13^c^ (1.12 – 1.15)	1.0 (0.99 – 1.01)	0.92^c^ (0.91 – 0.93)	0.95^c^ (0.94 – 0.96)	1.11^f^ (1.03 – 1.19)	1.05 (0.95 – 1.16)
**Charlson Comorbidity Index Score**						
0	Ref	Ref	Ref	Ref	NA	
1–3	2.46^c^ (2.44–2.48)	2.15^c^ (2.13 – 2.17)	1.17^c^ (1.16 – 1.18)	1.04^c^ (1.04 – 1.05)		
4+	4.37^c^ (4.32 – 4.41)	3.38^c^ (3.34 – 3.41)	1.17^c^ (1.15 – 1.21)	1.01 (0.99 – 1.04)		
Unknown	0.86^c^ (0.84 – 0.87)	0.94^c^ (0.93 – 0.96)	0.38^c^ (0.35 – 0.41)	0.42^c^ (0.39 – 0.45)		
**Pediatric Medical Complexity Algorithm Category**						
Not complex	NA				Ref	Ref
Non-Complex Chronic					1.47^c^ (1.4 – 1.55)	1.04 (0.99 – 1.1)
Complex chronic					3.61^c^ (3.5 – 3.72)	1.39^c^ (1.33 – 1.45)
**Vaccination**						
No evidence	Ref	Ref	Ref	Ref	Ref	Ref
Prior to index event	1.13^c^ (1.12 – 1.14)	0.98^c^ (0.97 – 0.98)	0.91^c^ (0.90 – 0.92)	0.84^c^ (0.83 – 0.85)	1.24^c^ (1.2 – 1.28)	1.1^c^ (1.05 – 1.15)
Close to index event	0.88^c^ (0.87 – 0.9)	0.97^c^ (0.95 – 0.98)	1.09^c^ (1.07 – 1.11)	1.12^c^ (1.10 – 1.14)	1.05 (0.99 – 1.11)	1.14^c^ (1.06 – 1.23)
**COVID-19 Severity**						
Not Hospitalized	Ref	Ref	Ref	Ref	Ref	Ref
Emergency Department	1.31^c^ (1.29 – 1.32)	1.21^c^ (1.2 – 1.22)	1.12^c^ (1.10 – 1.13)	1.09^c^ (1.08 – 1.10)	1.2^c^ (1.16 – 1.25)	1.15^c^ (1.09 – 1.21)
Hospitalized	3.14^c^ (3.11 – 3.17)	2.12^c^ (2.1 – 2.15)	1.6^c^ (1.58 – 1.62)	1.53^c^ (1.51 – 1.55)	3.78^c^ (3.55 – 4.03)	2.12^c^ (1.77 – 2.54)
Hospitalized with Intensive Care Unit-level care	3.84^c^ (3.76 – 3.91)	2.53^c^ (2.48 – 2.58)	2.41^c^ (2.35 – 2.48)	2.31^c^ (2.24 – 2.37)	5.26^c^ (4.42 – 6.25)	2.29^c^ (2.12 – 2.47)
Hospitalized and COVID-19 positive	1.99^c^ (1.96 – 2.02)	1.52^c^ (1.5 – 1.55)	1.92^c^ (1.89 – 1.95)	1.81^c^ (1.78 – 1.84)	4.12^c^ (4.0 – 4.24)	2.56^c^ (2.46 – 2.67)

a Hazard ratios generated by a Cox Proportional Hazards regression model, unadjusted.

b Hazard ratios generated by a multivariable Cox Proportional Hazards regression model, adjusted for age group, sex, race/ethnicity, pre-existing conditions, rurality, COVID-19 severity, vaccination status, and month of the index event.

c p-value <0.001

d p = 0.018

e p = 0.008

f p = 0.007

## Data Availability

See Supplement 2.

## Key Points

### Question:

What proportion of adults and children with known acute SARS-CoV-2 infection develop symptoms and conditions consistent with Long COVID within 180 days post-infection, and how has that proportion changed over time?

### Findings:

In this electronic health record-based study of COVID-19 positive patients across three national research networks, crude incidence of Long COVID, was 7% in children and ranged from 8.5% to 26.4% in adults, depending on approach used to detect Long COVID. Excess incidence of these symptoms or conditions was 4.2% in children and 7% in adults compared to uninfected individuals. Temporal patterns were consistent across networks.

### Meaning:

Findings indicate that preventing and mitigating Long COVID remains a public health priority.

## References

[1] Nalbandian A, Sehgal K, Gupta A, Post-acute COVID-19 syndrome. Nat Med. 2021;27(4):601–615. doi:10.1038/s41591-021-01283-z 33753937 PMC8893149

[2] Davis HE, McCorkell L, Vogel JM, Topol EJ. Long COVID: major findings, mechanisms and recommendations. Nat Rev Microbiol. 2023;21(3):133–146. doi:10.1038/s41579-022-00846-2 36639608 PMC9839201

[3] Soriano JB, Murthy S, Marshall JC, Relan P, Diaz JV, WHO Clinical Case Definition Working Group on Post-COVID-19 Condition. A clinical case definition of post-COVID-19 condition by a Delphi consensus. Lancet Infect Dis. 2022;22(4):e102–e107. doi:10.1016/S1473-3099(21)00703–9 34951953 PMC8691845

[4] HHS. COVID.gov - What is Long COVID. COVID.gov. Accessed November 17, 2023. https://www.covid.gov/longcovid/definitions

[5] Tartof SY, Malden DE, Liu ILA, Health Care Utilization in the 6 Months Following SARS-CoV-2 Infection. JAMA Netw Open. 2022;5(8):e2225657. doi:10.1001/jamanetworkopen.2022.25657 35960522 PMC9375168

[6] O’Mahoney LL, Routen A, Gillies C, The prevalence and long-term health effects of Long Covid among hospitalised and non-hospitalised populations: A systematic review and meta-analysis. EClinicalMedicine. 2023;55:101762. doi:10.1016/j.eclinm.2022.101762 36474804 PMC9714474

[7] Ledford H. How common is long COVID? Why studies give different answers. Nature. 2022;606(7916):852–853. doi:10.1038/d41586-022-01702-2 35725828

[8] Woodrow M, Carey C, Ziauddeen N, Systematic Review of the Prevalence of Long COVID. Open Forum Infect Dis. 2023;10(7):ofad233. doi:10.1093/ofid/ofad233 37404951 PMC10316694

[9] Pavli A, Theodoridou M, Maltezou HC. Post-COVID Syndrome: Incidence, Clinical Spectrum, and Challenges for Primary Healthcare Professionals. Arch Med Res. 2021;52(6):575–581. doi:10.1016/j.arcmed.2021.03.010 33962805 PMC8093949

[10] Bull-Otterson L, Baca S, Saydah S, Post–COVID Conditions Among Adult COVID-19 Survivors Aged 18–64 and ≥65 Years — United States, March 2020–November 2021. MMWR Morb Mortal Wkly Rep. 2022;71(21):713–717. doi:10.15585/mmwr.mm7121e1

[11] Sedgley R, Winer-Jones J, Bonafede M. Long COVID Incidence in a Large US Ambulatory Electronic Health Record System. Am J Epidemiol. 2023;192(8):1350–1357. doi:10.1093/aje/kwad095 37073410

[12] Fritsche LG, Jin W, Admon AJ, Mukherjee B. Characterizing and Predicting Post-Acute Sequelae of SARS CoV-2 Infection (PASC) in a Large Academic Medical Center in the US. J Clin Med. 2023;12(4):1328. doi:10.3390/jcm12041328 36835863 PMC9967320

[13] Menni C, Valdes AM, Polidori L, Symptom prevalence, duration, and risk of hospital admission in individuals infected with SARS-CoV-2 during periods of omicron and delta variant dominance: a prospective observational study from the ZOE COVID Study. The Lancet. 2022;399(10335):1618–1624. doi:10.1016/S0140-6736(22)00327-0 PMC8989396 35397851

[14] Antonelli M, Pujol JC, Spector TD, Ourselin S, Steves CJ. Risk of long COVID associated with delta versus omicron variants of SARS-CoV-2. The Lancet. 2022;399(10343):2263–2264. doi:10.1016/S0140-6736(22)00941-2 PMC9212672 35717982

[15] Thi Khanh HN, Cornelissen L, Castanares-Zapatero D, Association between SARS-CoV-2 variants and post COVID-19 condition: findings from a longitudinal cohort study in the Belgian adult population. BMC Infect Dis. 2023;23(1):774. doi:10.1186/s12879-023-08787-8 37940843 PMC10634063

[16] Diexer S, Klee B, Gottschick C, Association between virus variants, vaccination, previous infections, and post-COVID-19 risk. Int J Infect Dis. 2023;136:14–21. doi:10.1016/j.ijid.2023.08.019 37634619

[17] Willan J, Agarwal G, Bienz N. Mortality and burden of post-COVID-19 syndrome have reduced with time across SARS-CoV-2 variants in haematology patients. Br J Haematol. 2023;201(4):640–644. doi:10.1111/bjh.18700 36861893

[18] Long-covid symptoms are less common now than earlier in the pandemic. Washington Post. Published March 18, 2023. Accessed November 17, 2023. https://www.washingtonpost.com/health/2023/03/18/long-covid-less-likely/

[19] Du M, Ma Y, Deng J, Liu M, Liu J. Comparison of Long COVID-19 Caused by Different SARS-CoV-2 Strains: A Systematic Review and Meta-Analysis. Int J Environ Res Public Health. 2022;19(23):16010. doi:10.3390/ijerph192316010 36498103 PMC9736973

[20] Magnusson K, Turkiewicz A, Flottorp SA, Englund M. Prevalence of long COVID complaints in persons with and without COVID-19. Sci Rep. 2023;13(1):6074. doi:10.1038/s41598-023-32636-y 37055494 PMC10100609

[21] Hastie CE, Lowe DJ, McAuley A, True prevalence of long-COVID in a nationwide, population cohort study. Nat Commun. 2023;14(1):7892. doi:10.1038/s41467-023-43661-w 38036541 PMC10689486

[22] Haendel MA, Chute CG, Bennett TD, The National COVID Cohort Collaborative (N3C): Rationale, design, infrastructure, and deployment. J Am Med Inform Assoc. 2021;28(3):427–443. doi:10.1093/jamia/ocaa196 32805036 PMC7454687

[23] Pfaff ER, Madlock-Brown C, Baratta JM, Coding long COVID: characterizing a new disease through an ICD-10 lens. BMC Med. 2023;21(1):1–13. doi:10.1186/s12916-023-02737-6 36793086 PMC9931566

[24] Ukwishaka J, Ndayishimiye Y, Destine E, Danwang C, Kirakoya-Samadoulougou F. Global prevalence of coronavirus disease 2019 reinfection: a systematic review and meta-analysis. BMC Public Health. 2023;23(1):778. doi:10.1186/s12889-023-15626-7 37118717 PMC10140730

[25] Crosskey M, McIntee T, Preiss AJ, Reengineering a machine learning phenotype to adapt to the changing COVID-19 landscape: A study from the N3C and RECOVER consortia. Published online December 9, 2023:2023.12.08.23299718. doi:10.1101/2023.12.08.23299718

[26] Zang C, Zhang Y, Xu J, Data-driven analysis to understand long COVID using electronic health records from the RECOVER initiative. Nat Commun. 2023;14(1):1948. doi:10.1038/s41467-023-37653-z 37029117 PMC10080528

[27] Lorman V, Rao S, Jhaveri R, Understanding pediatric long COVID using a tree-based scan statistic approach: an EHR-based cohort study from the RECOVER Program. JAMIA Open. 2023;6(1):ooad016. doi:10.1093/jamiaopen/ooad016 36926600 PMC10013630

[28] Rao S, Lee GM, Razzaghi H, Clinical Features and Burden of Postacute Sequelae of SARS-CoV-2 Infection in Children and Adolescents. JAMA Pediatr. 2022;176(10):1000–1009. doi:10.1001/jamapediatrics.2022.2800 35994282 PMC9396470

[29] Zhang H, Zang C, Xu Z, Data-driven identification of post-acute SARS-CoV-2 infection subphenotypes. Nat Med. 2023;29(1):226–235. doi:10.1038/s41591-022-02116-3 36456834 PMC9873564

[30] CDC. CDC Museum COVID-19 Timeline. Centers for Disease Control and Prevention. Published March 15, 2023. Accessed November 17, 2023. https://www.cdc.gov/museum/timeline/covid19.html

[31] USDA ERS - Rural-Urban Commuting Area Codes. Accessed November 17, 2023. https://www.ers.usda.gov/data-products/rural-urban-commuting-area-codes/

[32] COVID-19 antivirals utilization: geographic and demographic patterns of treatment in 2022 - Digital Collections - National Library of Medicine. Accessed November 17, 2023. https://collections.nlm.nih.gov/catalog/nlm:nlmuid-9918590888406676-pdf

[33] Moynihan R, Sanders S, Michaleff ZA, Impact of COVID-19 pandemic on utilisation of healthcare services: a systematic review. BMJ Open. 2021;11(3):e045343. doi:10.1136/bmjopen-2020-045343 PMC7969768 33727273

[34] Stephens MD, Gazmararian JA, Khakharia A. Prevalence and Risk Factors of Post-Acute Sequelae of COVID-19 Among United States Veterans. Ann Epidemiol. Published online November 15, 2023:S1047–2797(23)00213–2. doi:10.1016/j.annepidem.2023.11.006 PMC10843577 37977283

[35] Global Burden of Disease Long COVID Collaborators. Estimated Global Proportions of Individuals With Persistent Fatigue, Cognitive, and Respiratory Symptom Clusters Following Symptomatic COVID-19 in 2020 and 2021. JAMA. 2022;328(16):1604–1615. doi:10.1001/jama.2022.18931 36215063 PMC9552043

[36] Brannock MD, Chew RF, Preiss AJ, Long COVID risk and pre-COVID vaccination in an EHR-based cohort study from the RECOVER program. Nat Commun. 2023;14(1):2914. doi:10.1038/s41467-023-38388-7 37217471 PMC10201472

[37] Brett KM, Burt CW. Utilization of ambulatory medical care by women: United States, 1997–98. Vital Health Stat 13. 2001;(149):1–46. doi:10.1037/e309022005-001 11478128

